# NiCo_2_S_4_ Nanotrees Directly Grown on the Nickel NP-Doped Reduced Graphene Oxides for Efficient Supercapacitors

**DOI:** 10.3390/ma12182865

**Published:** 2019-09-05

**Authors:** Wooree Jang, Won San Choi, Youn-Sik Lee, Hye Young Koo

**Affiliations:** 1 Functional Composite Materials Research Center, Korea Institute of Science and Technology (KIST), Jeonbuk Institute of Advanced Composite Materials, 92 Chudong-ro, Bongdong-eup, Wanju-gun, Jeollabuk-do 55324, Korea; 2 Department of Chemical and Biological Engineering, Hanbat National University, Daejeon 305-719, Korea; 3 School of Chemical Engineering, Chonbuk National University, Jeon-ju, Jeollabuk-do 54896, Korea

**Keywords:** nickel cobalt sulfide, reduced graphene oxide, nanocomposite, supercapacitor

## Abstract

In this work, we report a feasible fabrication of NiCo_2_S_4_ nanotree-like structures grown from the Ni nanoparticle (NP)-doped reduced graphene oxides (Ni-rGO) by a simple hydrothermal method. It is found that the presence of Ni NPs on the surface of the rGOs initiates growth of the NiCo_2_S_4_ nanotree flocks with enhanced interfacial compatibility, providing excellent cyclic stability and rate performance. The resulting NiCo_2_S_4_/Ni-rGO nanocomposites exhibit a superior rate performance, demonstrating 91.6% capacity retention even after 10,000 cycles of charge/discharge tests.

## 1. Introduction

Transition metal sulfides have great potential as active materials for high-performance supercapacitors. The use of binary or ternary metal sulfides enriches redox reaction sites that benefit from multiple valence states of various cationic species, leading to a higher specific capacitance and higher energy density. Among them, Nickel-Cobalt sulfide (NiCo_2_S_4_) species have been reported to exhibit high electrochemical activity and stability [1,2,3,4,5]. NiCo_2_S_4_ with various types of nanostructures were synthesized, demonstrating their excellent supercapacitive performances. However, application of these species as electrode materials is still limited due to their inferior rate capability and fast capacitance fading. To overcome these limitations, nanocomposites composed of the transition metal sulfides and conductive support materials such as nickel foam, carbon fiber, and porous carbons have been widely investigated [6,7,8,9]. Benefitting from the presence of the support structure, these nanocomposites could generally acquire enhanced conductivity and better electrochemical stability.

Up to now, many studies relevant to the nanocomposite of NiCo_2_S_4_ and rGO have been widely conducted [10,11]. Judging from most of the previous studies, the fabrication of hybrid nanostructures from the NiCo_2_S_4_ and conductive support materials often involves separate preparation of active and support materials and the subsequent anchoring of the both materials into hybrid nanostructures. This kind of process is found to be time-consuming and involves multi-step reactions.

In this work, we report the direct growth of nanotree-like NiCo_2_S_4_ structure on the surface of the Ni NP-doped reduced graphene oxides (Ni-rGOs) via a facile hydrothermal process. This is accomplished by the reaction of Ni and Co precursors and urea on the Ni-rGOs and subsequent heat treatment. Up to now, direct growth of the NiCo_2_S_4_ on the surface of rGOs using solution process was not being reported. We found that the vertical growth of the NiCo_2_S_4_ species with nanotree-like morphology could be realized by the growth initiated from the doped Ni NPs on a surface of the rGOs. Benefitting from the enhanced interfacial compatibility of the NiCo_2_S_4_ and Ni-rGOs, the resulting nanocomposites exhibit high specific capacitance with excellent cyclic stability of 91.6% capacity retention at 10,000 cycles of charge/discharge process.

## 2. Materials and Methods

### 2.1. Materials

H_2_SO_4_ (98%), H_2_O_2_ (35%), and HCl (5%) were purchased from Dae-Jung (Suwon, Korea). Graphite flakes, NaBH_4_, nickel (II) nitrate hexahydrate (Ni(NO_3_)_2_·6H_2_O), cobalt (II) nitrate hexahydrate (Co(NO_3_)_2_·6H_2_O), urea, thiourea, ammonium fluoride (NH_4_F), KOH, N-methyl-2-pyrrolidone (NMP), and polyvinylidene fluoride (PVDF) were purchased from Sigma-Aldrich (Steinheim, Germany). All of these materials were used without any further purification.

### 2.2. Preparation of GO

A modified Hummers’ method was used to prepare the Graphite oxide (GO), as described in previous reports [12]. Briefly, 2 g of graphite flakes were mixed with 46 mL of 98% sulfuric acid in a 250 mL round bottom flask and placed in an ice-bath with constant stirring. Then 6 g of potassium permanganate was slowly added to the mixture. After 2 h, the supernatant mixture was transferred to an oil-bath and kept at a constant temperature a 35 °C for 6 h. Then 92 mL of de-ionized (DI) water was gradually added to the reaction mixture. The mixture was then stirred for 1 h. The whole reaction mixture was then poured into a 1 L beaker containing 240 mL of water. Then 35% hydrogen peroxide solution was added until the color of the mixture changed to bright yellow. Hydrochloric acid diluted in 5% water was added in order to remove the metal cations. Finally, the resulting solution was washed with DI water, and dialysis was performed until the neutral graphite oxide solution was obtained. 

### 2.3. Preparation of Ni-Doped rGOs (Ni-rGO) 

A measure of 50 mg of GO was dispersed in 50 mL of NMP using water bath sonication for 2 h. Then 100 mg of Ni(NO_3_)_2_·6H2O was dissolved in 20 mL NMP by vigorous stirring at 80 °C. The solution color was changed brown to black after slowly adding 20 mg of NaBH_4_ dissolved in 10 mL NMP. This mixture was poured into the brown GO solution and sonicated for 2 h. After sonication, the black mixed solution could be obtained. The solution was washed several times by ethanol and water. 

### 2.4. Growth of NiCo_2_S_4_ Nanotrees the Ni Doped-rGO (NiCo_2_S_4_/Ni-rGO) 

25 mg of the as-obtained sample was dissolved in 30 mL of DI water. Then 3 mM of Co(NO_3_)_2_·6H_2_O, 3 mM of Ni(NO_3_)_2_·6H_2_O, 17 mM of urea powder, and 4.5 mM of NH_4_F were added and then sonicated for 10 min. The mixed solution was poured into a 50 mL Teflon-lined stainless steel autoclave and heated at 150 °C for 4 h, and then the solution was washed for several times. Finally, 10 mg of the as-collected sample was dissolved in DI water (30 mL), and 17 mM of thiourea powder was added to the solution. The resulting mixture were heated at 180 °C for 6 h in an autoclave. The final product was washed with DI water, ethanol, and subsequently freeze-dried to achieve nanotree-like NiCo_2_S_4_/Ni-rGO nanocomposites.

### 2.5. Characterization

X-ray diffraction (XRD) patterns were recorded using a Rigaku SmartLab diffractometer (Tokyo, Japan), and using Cu Kα radiation. X-ray photoelectron spectroscopy (XPS) spectra were obtained by using a Thermo Fisher Scientific (Waltham, MA, USA) K-alpha with an Al source. The field emission scanning electron microscope (FE-SEM) images and energy dispersive spectroscopy (EDS) analysis were performed using a Verios 460L (FEI, Hillsboro, OR, USA). Transmission electron microscopy (TEM) images were taken by a Tecnai G2 F20 (FEI, Hillsboro, OR, USA).

### 2.6. Electrochemical Measurements

Electrochemical measurements were performed using a 6 M KOH solution in a three-electrode cell on a Zive SP1 electrochemical working station (CH Instruments, Inc., Austin, TX, USA). Platinum wire and Hg/HgO electrodes were used as the counter electrode and the reference electrodes, respectively. The as-prepared product was mixed with 15 wt % acetylene black, 5 wt % PVDF, and a small amount of NMP to produce a homogeneous paste, which was applied onto a nickel form. The electrode was then heated at 80 °C for 12 h to evaporate the solvent. The rate of total loading mass was 1 mg cm^−2^.

## 3. Results and Discussion

### 3.1. Materials Characterization

**Figure 1:**
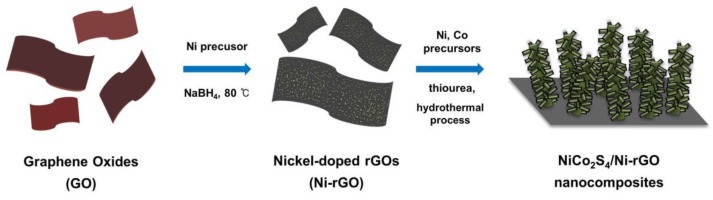
Schematic illustration for the preparation of the NiCo_2_S_4_/Ni-rGO nanotree-like composites.

**Figure 2:**
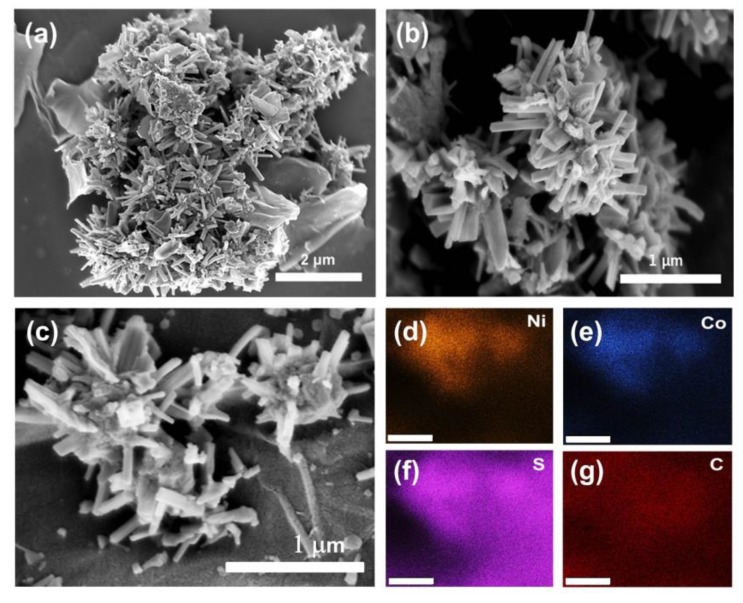
(**a**) Scanning electron microscopic images of the nanotree-like NiCo_2_S_4_/Ni-rGO composites; (**b**) magnified image; (**c**) selected area SEM image for EDS mapping; (**d**–**g**) elemental distribution of Ni, Co, S, and C from the SEM image (**c**) (scale bar: 500 nm).

The overall schematic for preparation of the NiCo_2_S_4_/Ni-rGO nanocomposite is presented in Figure 1. Firstly, nickel is doped on the surface of the rGOs for growth of the NiCo_2_S_4_ nanotree-like structures. By subsequent hydrothermal process with Ni and Co precursors and urea, NiCo_2_S_4_/Ni-rGO hybrid nanocomposites were successfully synthesized. Figure 2 shows typical scanning electron microscope (SEM) images for the synthesized NiCo_2_S_4_/Ni-rGOs. As seen in Figure 2a, each NiCo_2_S_4_ nanorods were densely congregated as a flock of tree branch-like structures. The length of each NiCo_2_S_4_ nanorod is in the range of several hundred nanometers, as shown in Figure 2b. These tree-like NiCo_2_S_4_ nanostructures were found to be vertically grown from the Ni-doped rGOs. From Figure 2d to Figure 2g displays the SEM-EDS maps showing the distributions of Ni, Co, S, and C acquired from Figure 2c. They demonstrate uniform distribution of Ni, Co, and S species in the nanotrees grown from the surface of the C-abundant Ni-rGOs. The atomic percentages of Ni, Co, S, and C were 4.18%, 1.95%, 29.74%, and 64.13%, respectively. (See Supplementary Materials Figure S1 for characterizations of the Ni-rGOs.)

**Figure 3:**
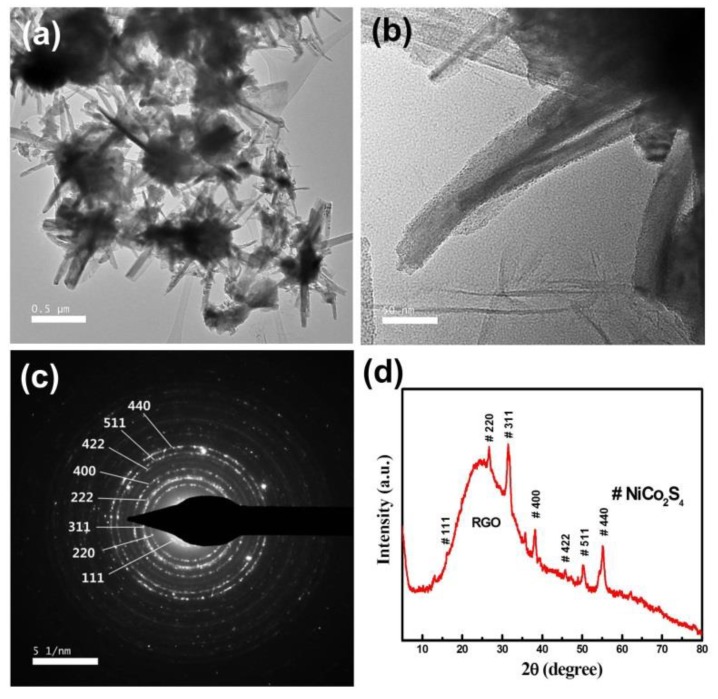
(**a**) Typical Transmission electron microscopic image of the NiCo_2_S_4_/Ni-rGO nanocomposites; (**b**) enlarged image of one branch; (**c**) SAED pattern of (**b**); (**d**) XRD pattern of the NiCo_2_S_4_/Ni-rGO nanocomposites.

To obtain detailed information on the microstructure of the NiCo_2_S_4_/Ni-rGO structure, TEM imaging analyses were conducted. Figure 3a shows typical image of the NiCo_2_S_4_/Ni-rGO nanocomposites, exhibiting flocks of nanorods grown vertically from the rGO surfaces. The magnified image of the nanocomposite shows the multiple packing of nanorods, with diameters of 5–10 nm (Figure 3b). The selected area electronic diffraction pattern (SAED) of the nanocomposites in Figure 3b shows well-defined rings, revealing NiCo_2_S_4_ on the surface of Ni-rGOs (Figure 3c). The rings can be indexed to cubic NiCo_2_S_4_. The crystal structure of the NiCo_2_S_4_/Ni-rGO is confirmed by X-ray diffraction (XRD) analysis (Figure 3d). A broad peak located around 24° is from presence of the rGOs. The characteristic peaks were appeared at 17.2°, 26.7°, 31.5°, 38.2°, 47.5°, 50.2°, and 55.3°, indicating (111), (220), (311), (400), (422), (511), and (440) planes of the NiCo_2_S_4_, respectively. These peaks were indexed as standard NiCo_2_S_4_ with a cubic phase (Joint Committee on Powder Diffraction Standards, JCPDS No.20-0782), and this agrees well with the SAED results. 

**Figure 4:**
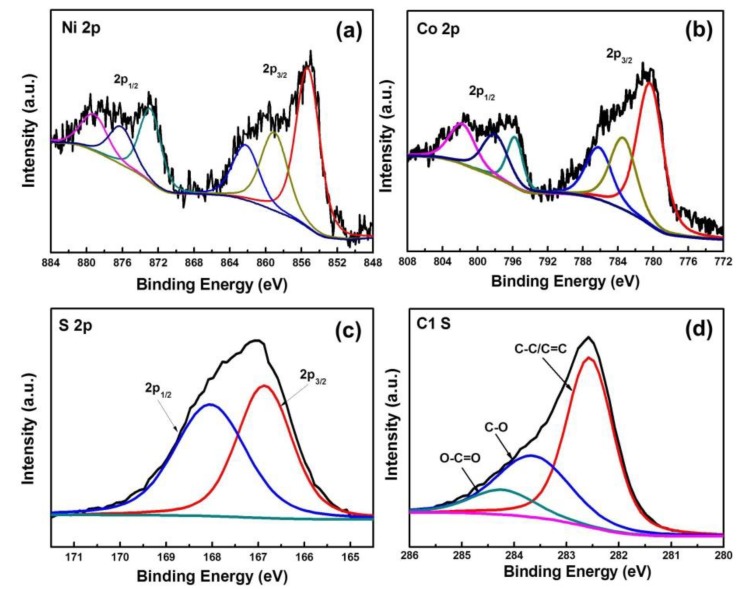
X-ray photoelectron spectroscopic spectra of the NiCo_2_S_4_/Ni-rGO nanocomposites: (**a**) Ni 2p; (**b**) Co 2p; (**c**) S 2p; and (**d**) C 1s spectra.

Detailed analyses on the chemical composition of the NiCo_2_S_4_/Ni-rGO were undertaken by X-ray photoelectron spectroscopy (XPS) measurements [13]. The resulting survey spectra of the NiCo_2_S_4_/Ni-rGO contain elements such as Ni, Co, S, and C (Figure S2). Figure 4a shows the Ni 2p spectrum with two spin-orbit doublet peaks of Ni 2p_3/2_ and Ni 2p_1/2_. The peaks at 855.29 eV and 872.88 eV were attributed to the presence of Ni^2+^, and the peaks at 859.03 eV and 876.11 eV were due to the Ni^3+^, suggesting coexistence of the Ni^2+^ and Ni^3+^ states in the NiCo_2_S_4_/Ni-rGO. Figure 4b shows the Co 2p spectrum with two peaks of Co 2p_3/2_ and Co 2p_1/2_ and with the shake-up satellite peaks. The first doublet at 777.9 eV and 793.0 eV and the second doublet at 780.6 and 797.2 eV were due to the presence of Co^3+^ and Co^2+^, respectively. The peaks from S 2p spectrum centered at 166.87 eV and 168.04 eV can be assigned to S 2p_3/2_ and S 2p_1/2_, respectively (Figure 4c) [14]. Figure 4d shows the C 1s spectrum having a major peak at 282.5 eV, which is in accordance with a previous report on the XPS spectrum of graphene nanosheets with C=C, C-O, and O=C-O bonds [15]. Due to the doped Ni NPs on the surface of the rGOs, the C1s peaks of the NiCo_2_S_4_/Ni-rGO were found to be a bit shifted to a lower binding energy.

It is found that the presence of the doped Ni NPs on the surface of the rGOs is crucial for the direct vertical growth of the NiCo_2_S_4_ nanotrees from the rGOs. To identify the morphological difference in the microstructure, identical hydrothermal growth of the NiCo_2_S_4_ was conducted on the surface of rGOs without Ni doping. From XRD studies, it is found that the peak positions of the resulting NiCo_2_S_4_ are almost same with that of the NiCo_2_S_4_/Ni-rGOs (Figure S3a). However, morphology of the resulting NiCo_2_S_4_/rGO is quite different than that of the NiCo_2_S_4_/Ni-rGOs. By examining the SEM image, we can find the presence of random NiCo_2_S_4_ polycrystallines without any well-defined nanotree morphology, and it looks like there is no systematic formation of interfacial conjunction with rGO nanosheets (Figure S3b). 

### 3.2. Electrochemical Properties

**Figure 5:**
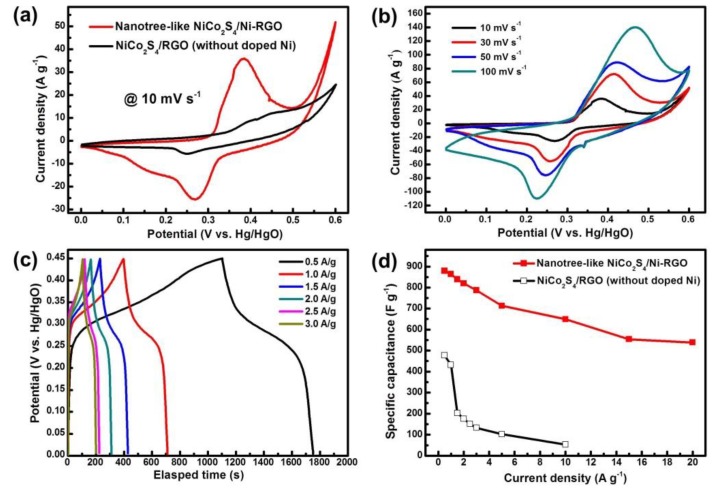
Electrochemical characterization of the NiCo_2_S_4_/Ni-rGO nanocomposites: (**a**) Cyclic voltammetric curves with/without Ni-doping; (**b**) CV curves at various scan rates; (**c**) Galvanostatic charge-discharge curves at different current densities; and (**d**) specific capacitance vs. current density plots.

To evaluate the electrochemical performance of the NiCo_2_S_4_/Ni-rGO nanocomposites, we performed cyclic voltammetry (CV), galvanostatic charge-discharge (GCD), and electrochemical impedance studies in a 6 M KOH electrolyte at various scan rates and current densities using a three electrode system. The results from CV measurements are displayed in Figure 5a. The nanocomposites showed a pair of redox peaks originating from the Faradaic reactions that was relevant to the Co^2+^/Co^3+^ and the Ni^2+^/Ni^3+^ redox couples based on the following reactions [16,17,18].

CoS + OH^−^ ⇌ CoSOH + e^−^

CoSOH + OH^−^ ⇌ CoSO + H2O + e^−^

NiS + OH^−^ ⇌ NiSOH + e^−^

However, in comparison with the NiCo_2_S_4_/rGO nanocomposite without the doping of Ni ions, the nanotree-like NiCo_2_S_4_/Ni-rGO nanocomposite exhibits a larger capacitive area at the same scan rate of 10 mV·s^−1^. We speculate that the vertical nanotree-like morphology of the NiCo_2_S_4_/Ni-rGO may induce the formation of the intact interface of NiCo_2_S_4_ and rGOs, providing an electrochemically enriched active site and an efficient transport path of electrons and ions capable of better capacitance performance compared to that of the NiCo_2_S_4_/rGOs. The CV analysis of nanotree-like NiCo_2_S_4_/Ni-rGO at different scan rates was performed to investigate the current response of the nanocomposite (Figure 5b). As the scan rate increased, the CV area also increased by retaining its shape, and the peak current density increased with increasing scan rates suggesting rapid Faradaic reaction. Also, the anodic and cathodic peaks were shifted towards positive and negative potential sides, due to the polarization of the electrode at higher scan rates [19,20,21]. Figure 5c exhibits the GCD results of the NiCo_2_S_4_/Ni-rGO with Faradaic reaction type performance and with a distinct plateau in the charging and discharging process. The specific capacitance values of the NiCo_2_S_4_/Ni-rGO composites were calculated from the following Equation (1):

(1) $$\mathrm{Cs}=\;\frac{2i_{m}\int^{\text{​}}V\;dt}{V^{2}|\begin{array}{c} Vf\\ Vi \end{array}}$$

where C_s_ (F·g^−1^) is the specific capacitance, *i_m_* = *I/m* (A g^−1^) is the current density, I is the current, *m* is the mass of active materials, $\int^{\text{​}}Vdt$ is the integral current area, and *V* is the potential with initial and final values of *V_i_* and *V_f_*, respectively [22]. Figure 5d demonstrated the calculated C_s_ of the NiCo_2_S_4_/Ni-rGO in comparison with the NiCo_2_S_4_/rGO at various current densities. The highest and the lowest C_s_ values of the NiCo_2_S_4_/Ni-rGO were 879.01 F·g^−1^ and 538.67 F·g^−1^ at current densities of 0.5 A·g^−1^ and 20 A·g^−1^, respectively. Comparatively, the highest and the lowest C_s_ of the NiCo_2_S_4_/rGO were 477.06 F·g^−1^ and 52.78 F·g^−1^ at current densities of 0.5 A·g^−1^ and 10 A·g^−1^, respectively. In agreement with the CV results from Figure 5a, the nanotree-like NiCo_2_S_4_/Ni-rGO composites showed better capacitance than NiCo_2_S_4_/rGO.

**Figure 6:**
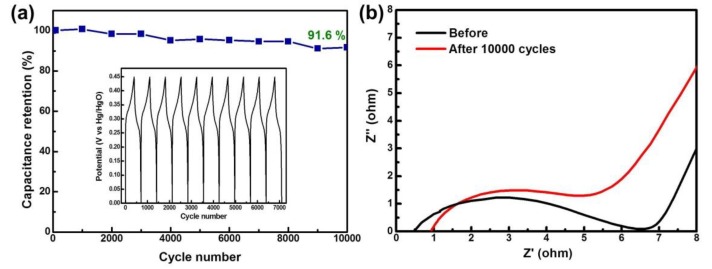
(**a**) Cyclic stability of the NiCo_2_S_4_/Ni-rGO nanocomposites at a current density of 1.0 A·g^−1^; (**b**) Nyquist plots of the NiCo_2_S_4_/Ni-rGO nanocomposites before and after cycling of 10,000 cycles.

In addition, we find that the doped Ni NPs on the surface of the rGOs helps to enhance the cyclic stability of the NiCo_2_S_4_/Ni-rGO nanocomposites at a high current density. Figure 6a presents the cyclic stability results of the NiCo_2_S_4_/Ni-rGO composites tested at 1 A·g^−1^ using a 6 M KOH solution in a three-electrode cell. Platinum wire and Hg/HgO electrodes were used as the counter electrode and the reference electrodes, respectively. Even after tests of 10,000 charge/discharge cycles, specific capacitance was retained as 91.6% (see Figure S4 for comparative performance of similar electrode materials). This enhanced cyclic stability can be attributed to the enhanced interfacial compatibility between the vertically grown NiCo_2_S_4_ and the rGOs thanks to the doped Ni ions, with increased conductivity from the contributions of rGOs [23]. Figure 6b displays Nyquist plots of the NiCo_2_S_4_/Ni-rGO before and after 10,000 cycles of charge/discharge in the frequency range of 0.01 Hz to 100 kHz. The lower value of the equivalent series resistance (ESR) is related to the conductivity of the electrolyte, the internal resistance of the electrode, and the contact resistance between the electrode and the electrolyte. ESR values before and after 10,000 cycles were measured at 0.51 and 0.937, respectively, providing additional evidence of the good electrochemical cyclic stability of the NiCo_2_S_4_/Ni-rGO. The difference in the initial resistances before and after cycling is due to the difference of the solution resistance (R_s_) of the NiCo_2_S_4_/Ni-rGO materials. It is known that electrochemical activity is unavoidably degraded during the long-term GCD cycles. Probable structural degradation after repetitive GCD cycles leads to loss of electrical conductivity at the electrode interface, contributing to increased R_s_ in the EIS spectra. In addition, a slight increase of the semicircle in the high-frequency range suggests a possible increase in a charge transfer resistance (R_ct_) after a test of 10,000 cycles [24]. Based on the results of CV, GCD, and EIS analysis, we conclude that the NiCo_2_S_4_/Ni-rGO nanocomposite is an effective material for use as a supercapacitor electrode. 

## 4. Conclusions

Nanotree-like NiCo_2_S_4_/Ni-rGO composites were synthesized by a facile hydrothermal growth of the NiCo_2_S_4_ on the surface of the Ni NP-doped rGOs. The Ni NPs on the surface of the rGOs help direct the growth of NiCo_2_S_4_ with nanotree-like morphology, enhancing capacitance properties by promoting interfacial compatibility of the NiCo_2_S_4_ and rGOs. The resulting NiCo_2_S_4_/Ni-rGO nanocomposite exhibited a specific capacitance of 879.01 F·g^−1^ at a current density of 0.5 A·g^−1^. In addition, it provided excellent capacitance retention of 91.6% even after 10,000 cycles of repetitive charge/discharge. The enhanced cyclic stability of the NiCo_2_S_4_/Ni-rGOs is believed to be related to a directly grown structure capable of providing rapid electron/ion transport as well as enhanced electrical conductivity. This kind of nanocomposite can be used as efficient electrode materials for various energy applications.

## Supplementary Materials

The following are available online at https://www.mdpi.com/1996-1944/12/18/2865/s1, Figure S1: Characterizations of the Ni-rGOs, Figure S2: XPS survey scan of the NiCo_2_S_4_/Ni-rGO nanocomposites, Figure S3: Characterizations of the NiCo_2_S_4_/rGO nanocomposites without doping of Ni ions on the surface of the rGOs.
