# Supplementary material for: NiCo2S4 Nanotrees Directly Grown on the Nickel NP-Doped Reduced Graphene Oxides for Efficient Supercapacitors
Source: Materials (Basel). 2019 Sep 5;12(18):2865. doi: 10.3390/ma12182865 (PMC6766062; doi:10.3390/ma12182865)
Supplement: Supplementary file 1 [file materials-12-02865-s001.pdf]

materials

MDPI

Article

$NiCo_2S_4$ Nanotrees Directly Grown on the Nickel NP-Doped Reduced Graphene Oxides for Efficient Supercapacitors

Wooree Jang [1], Won San Choi [2] and Youn-Sik Lee [3,*] and Hye Young Koo [1,*]

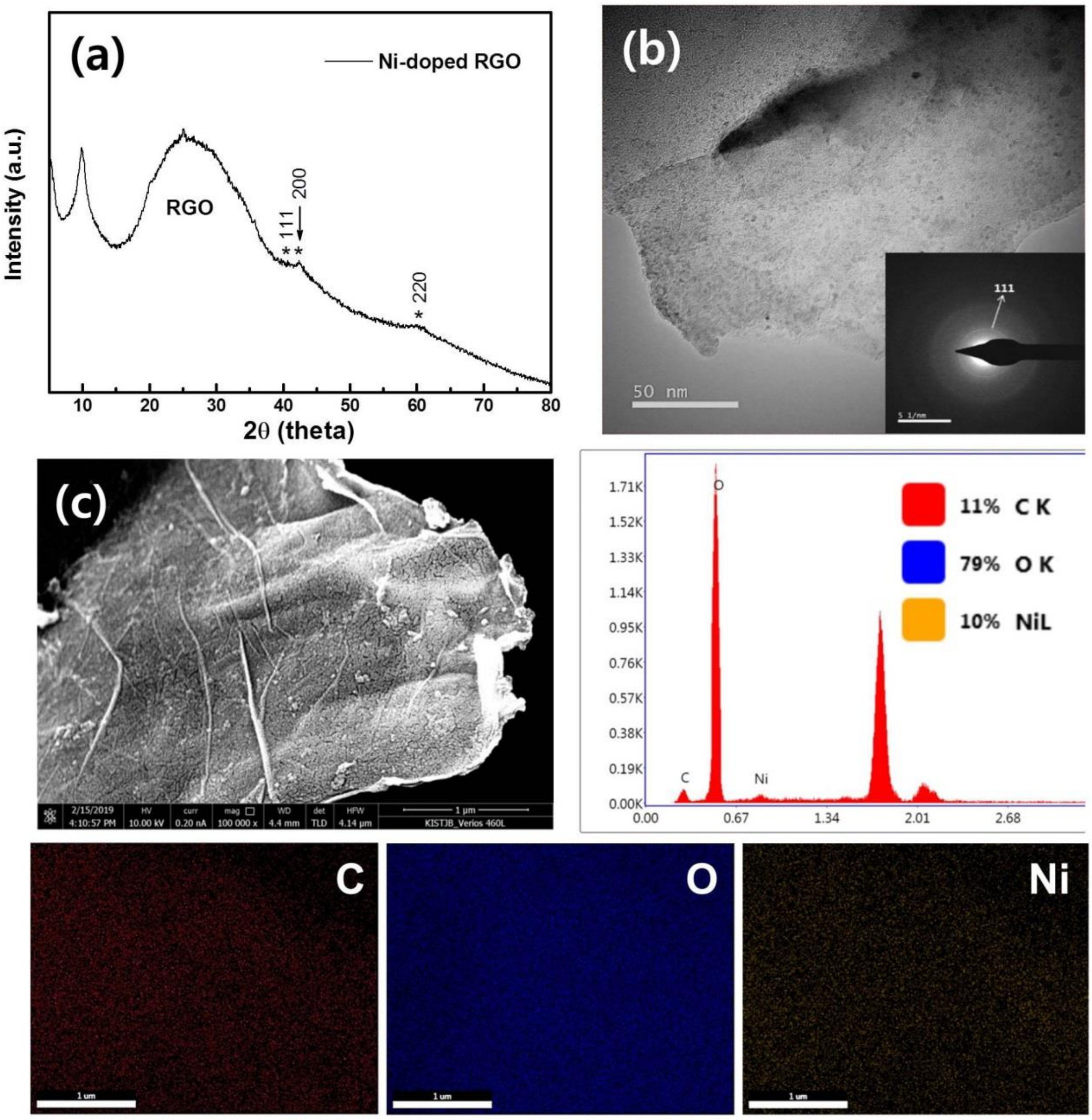

Figure S1. Characterizations of the Ni-rGOs. (**a**) XRD spectrum; (**b**) TEM image; (**c**) SEM image and corresponding EDS mapping of element C, O, and Ni results. XRD spectrum indicates the (200) crystal planes of cubic Ni lattice, and nominal (111) and (220) crystal planes of NiO lattices. The TEM and SEM images revealed the presence of Ni particles with uniform sized and well dispersed on the rGOs. The EDS mapping also shows uniform distributions of Ni on the surface of the rGOs.

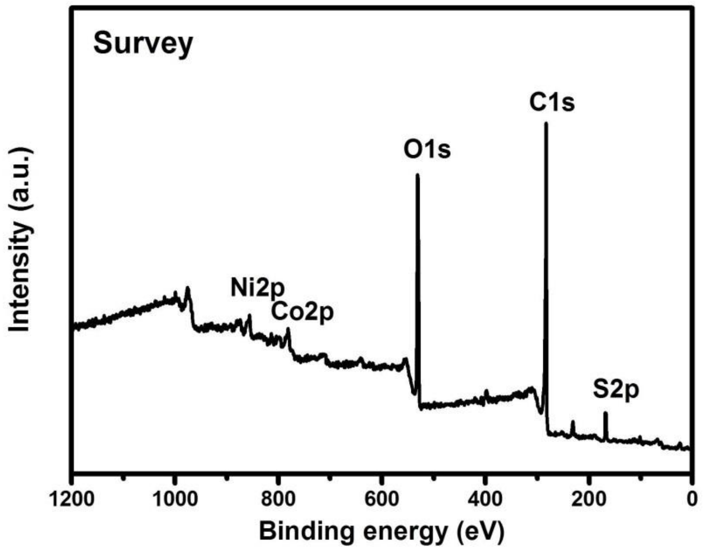

Figure S2. XPS survey scan of the $NiCo_2S_4$/Ni-rGO nanocomposites.

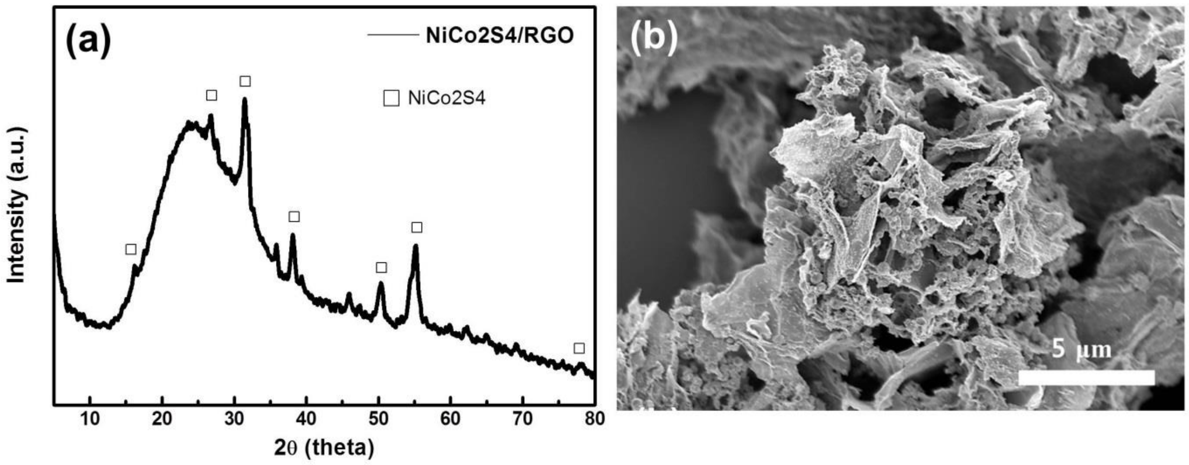

Figure S3. Characterizations of the $NiCo_2S_4$/rGO nanocomposites without doping of Ni ions on the surface of the rGOs. (**a**) XRD spectrum; (**b**) SEM image.

Composite material	Specific capacitance (F g^{-1})	Current density (A g^{-1})	Cycle life	Reference
NiCo2S4@rGO@ CNT composite	1242	2	54% after 2000 cycles	[1]
NiCo2S4 crystal nanostructure	835	1	92.7% after 5,000 cycles	[2]
NiCo2S4-rGO porous composite	1107	1	95% after 8,000 cycles	[3]
NiCo2S4 caterpillar-like nanostructures	1777	1	83% after 3,000 cycles	[4]
Co3O4@CoS on carbon	764	1	78.1% after 5,000 cycles	[5]
NiCO2S4 nanosheets on graphene	1220	1	91% after 2,000 cycles	[6]
NiCo2S4/rGO nanotrees	860	1	91.6% after 10,000 cycles	This work

Figure S4. Performance comparison of the $NiCo_2S_4$/Ni-rGO nanocomposite electrode materials with previous works based on similar materials.

References

1. Xu, J.; Yang, Y.; Chu, H.; Tang, J.; Ge, Y.; Shen, J.; Ye, M. Novel $NiCo_2S_4$@reduced graphene oxide@carbon nanotube nanocomposites for high performance supercapacitors. *RSC Adv*. **2016**, *6*, 100504.
2. Hussain, S.; Liu, T.; Aslam, N.; Zhang, Y.; Zhao, S. Truncated $NiCo_2S_4$ cobohexa-octahedral nanostructures for high-performance supercapacitor. *Mater. Lett*. **2017**, *189*, 21.
3. Fan, Y.-M.; Liu, Y.; Liu, X.; Liu, Y.; Fan, L.-Z. Hierarchical porous $NiCo_2S_4$-rGO composites for high-performance supercapacitors. *Electrochimica Acta* **2017**, *249*, 1.
4. Chen, X.; Chen, D.; Guo, X.; Wang, R.; Zhang, H. Facile growth of caterpillar-like $NiCo_2S_4$ nanocrystal arrays on nickel foam for high-performance supercapacitors. *ACS Appl. Mater. Inter*. **2017**, *9*, 18774.
5. Ning, J.; Zhang, T.; He, Y.; Jia, C.; Saha, P.; Cheng, Q. Co_3O_4@CoS core-shell nanosheets on carbon cloth for high performance supercapacitor electrodes. *Materials* **2017**, *10*, 608.
6. Peng, S.; Li, L.; Li, C.; Tan, H.; Cai, R.; Yu, H.; Mhaisalkar, S.; Srinivasan, M.; Ramakrishna, S.; Yan, Q. *In situ* growth of $NiCo_2S_4$ nanosheets on graphene for high-performance supercapacitors. *Chem. Commun.* **2013**, *49*, 10178.

© 2019 by the authors. Submitted for possible open access publication under the terms and conditions of the Creative Commons Attribution (CC BY) license (http://creativecommons.org/licenses/by/4.0/).
